# How the Color Fades From *Malus halliana* Flowers: Transcriptome Sequencing and DNA Methylation Analysis

**DOI:** 10.3389/fpls.2020.576054

**Published:** 2020-09-23

**Authors:** Mei-Ling Han, Jiao Yin, Yu-Heng Zhao, Xue-Wei Sun, Jia-Xin Meng, Jing Zhou, Ting Shen, Hou-Hua Li, Fan Zhang

**Affiliations:** ^1^College of Landscape Architecture and Art, Institute of Ornamental Plants, Northwest A&F University, Yangling, China; ^2^Sanqin Institute of Botany, Shaanxi Qincao Ecological Environment Technology Co., Ltd., Xi’an, China

**Keywords:** flower color, anthocyanin, methylation, MYB10, transcriptome sequencing, *Malus halliana*

## Abstract

The flower color of many horticultural plants fades from red to white during the development stages, affecting ornamental value. We selected *Malus halliana*, a popular ornamental species, and analyzed the mechanisms of flower color fading using RNA sequencing. Forty-seven genes related to anthocyanin biosynthesis and two genes related to anthocyanin transport were identified; the expression of most of these genes declined dramatically with flower color fading, consistent with the change in the anthocyanin content. A number of transcription factors that might participate in anthocyanin biosynthesis were selected and analyzed. A phylogenetic tree was used to identify the key transcription factor. Using this approach, we identified *MhMYB10* as directly regulating anthocyanin biosynthesis. *MhMYB10* expression was strongly downregulated during flower development and was significantly positively related to the expression of anthocyanin biosynthetic genes and anthocyanin content in diverse varieties of *Malus*. To analyze the methylation level during flower development, the *MhMYB10* promoter sequence was divided into 12 regions. The methylation levels of the R2 and R8 increased significantly as flower color faded and were inversely related to *MhMYB10* expression and anthocyanin content. Therefore, we deduce that the increasing methylation activities of these two regions repressed *MhMYB10* expression.

## Introduction

**Figure 1:**
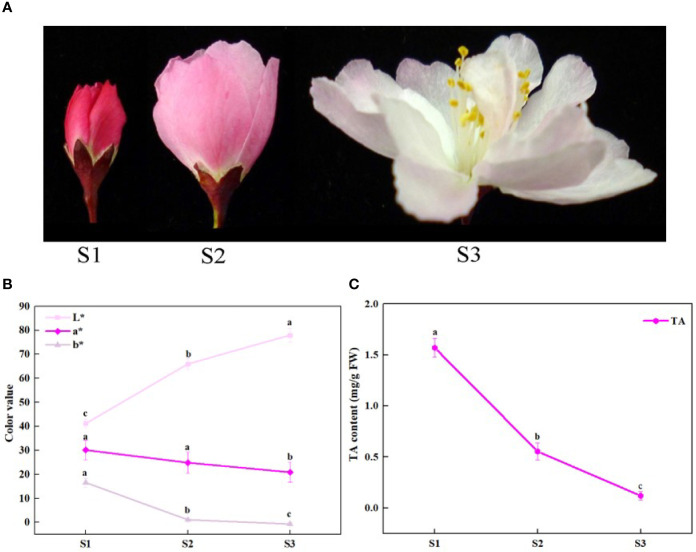
Schematic of phenotypic and total anthocyanin content at different developmental stages of *Malus halliana*. **(A)** The flower color change at three stages. **(B)** L*a*b* values of petals at three stages. **(C)** The total anthocyanin (TA) content at three stages. S1, small bud stage; S2, initial-flowering stage; S3, late-flowering stage. Error bars indicate standard deviation (SD) obtained from four biological replicates. Different letters between cultivars denote significant differences (Duncan test, *p* < 0.05).

*Malus halliana* is a traditional and important ornamental plant in the Rosaceae family because of its elegant flower shape and gorgeous flower color. *M. halliana* is also valued for its profound cultural heritage and broad pharmaceutical value in China (Cui et al., 2018). Flower color is a major characteristic of ornamental value, but the petal color of *M. halliana* clearly changes from red to pale pink during flower development (**Figure 1A**), which affects its ornamental value. This fading of flower color is widespread in *Malus* plants and other ornamental plants (Jiang et al., 2014; Yue et al., 2019). The phenotype change of flower color of *M. halliana* presents distinct visible phases, so *M. halliana* is considered as an excellent model plant for research on flower color.

A number of authors have reported that the contents and concentrations of anthocyanins play crucial roles in flower color formation (Alessandra and Luca et al., 2013; Qin et al., 2018). Anthocyanins, a class of plant flavonoid metabolites, are synthesized through a number of enzymes, which catalyze sequential reactions for anthocyanin biosynthesis within the cytosol compartment (Xie et al., 2014). Firstly, phenylalanine is converted to coumarate-CoA by phenylalanine ammonia lyase (PAL), cinnamate-4-hydroxylase (C4H), and 4-coumarate-CoA ligase (4CL). Secondly, coumarate-CoA is catalyzed to flavanone by chalcone synthase (CHS) and chalcone isomerase (CHI). The formation of various dihydroflavonols is catalyzed by flavanone 3-hydroxylase (F3H) and flavonoid-3’-monooxygenase (F3’H). Thirdly, the anthocyanidins are synthesized by dihydroflavonol 4-reductase (DFR) and anthocyanidin synthase (ANS). Finally, the synthesized anthocyanidins are unstable and modified through a series of glycosylation to form stable anthocyanins catalyzed by UDP-glucose transferase enzymes (UGT) (Zhao and Tao, 2015). The expression of most genes encoding for these enzymes is positively associated with anthocyanin content in *Malus* spp. (Honda et al., 2002; Xie et al., 2014; Tian et al., 2015). Comparative transcriptomic of *Malus* spp. reveals that the *ANS*, *4CL*, and *FGT* genes in the anthocyanin biosynthetic pathway are highly expressed in red petals (Huang et al., 2020).

The anthocyanin biosynthetic pathway is controlled by a transcription complex composed of three transcription factors (TFs): R2R3-MYB, bHLH, and WD40 protein. The MYB family is the important TFs in anthocyanin biosynthetic pathway; MYBs are divided into 28 subgroups (Sg, from Sg1 to Sg28) based on conserved amino acid sequence domains in C terminal, and Sg6 was identified as directly regulating anthocyanin biosynthesis (Stracke et al., 2001; Hong et al., 2015). MYB10, which belongs to Sg6, performs a key regulating function in anthocyanin biosynthesis through binding to the promoter of anthocyanin biosynthetic structural genes in *Malus domestica*, *Pyrus* spp., and *Amygdalus persica* (Espley et al., 2007; Feng et al., 2010; Zhou et al., 2019). Moreover, researchers have reported that other TFs, such as WRKY, ethylene response factors (ERF), NAC, zinc finger, and MADS-box proteins, are involved in the anthocyanin biosynthetic pathway in *Pyrus* spp., *Litchi chinensis*, *Arabidopsis*, and *Brassica napus* (Duan et al., 2018; Ni et al., 2018; Jiang et al., 2019). However, there has been little research addressing how genetic information and transcription contribute to the regulation of flower color in *Malus* spp.

Recent studies have demonstrated that the anthocyanin biosynthetic pathway is controlled by epigenetic mechanisms. A major and conserved epigenetic mechanism in regulating gene expression is DNA methylation of the gene promoter (Huang et al., 2019). Liu et al. (2012) reported that the failure of anthocyanin accumulation in floral tissues of *Oncidium* spp. might be attributed to expression inhibition of *CHS* because of the methylation of the promoter sequence. Diverse methylation on the *MYB10* promoter was found to be the key reason for fruit color formation (Telias et al., 2011; Wang et al., 2017). However, research on the effect of promoter methylation of the transcriptional factor on flower color formation during development remains unclear.

In addition to biosynthesis, the process of anthocyanin transport from the endoplasmic reticulum and accumulation of pigmentation in the vacuole is necessary for tissue coloring and is catalyzed by corresponding enzymes: glutathione S-transferase (GST) family, the ATP-binding cassette (ABC) and multidrug and toxic compound extrusion (MATE) families (Mueller et al., 2000; Klein et al., 2006; Gomez et al., 2009). Recently, much attention has also been directed to the relationship of color fading and anthocyanin degradation involved in three common enzyme families, including polyphenol oxidases (PPOs), class III peroxidases, and intracellular laccase (Oren-Shamir, 2009; Fang et al., 2015). However, in contrast to the detailed knowledge regarding anthocyanin biosynthesis, little is known about degradation of these pigments.

To understand the mechanism of flower color fading in *M. halliana* in this study, the petals at different flowering stages were analyzed using RNA sequencing. A set of differentially expressed genes (DEGs) potentially involved in flower coloration was identified. The methylation in the *MhMYB10* promoter was also analyzed, which revealed the dynamic mechanism of methylation during flower color formation. This study will help to advance knowledge of the temporal and spatial regulation of anthocyanin biosynthesis in *M. halliana*, which underlies the diverse flower color intensities and patterning in *Malus* spp.

## Materials and Methods

### Plant Materials

*Malus* spp., including *M. halliana*, *M*. ‘Pinkspires’, *M*. ‘Strawberry Parfait’, and *M*. ‘Snowdrift’, grown in the crabapple resource nursery of Northwest A&F University, Yangling, China, were used as plant materials. The flowers were collected from healthy and approximately uniform trees from March to April 2018. Sepals, pistils, and stamens were quickly removed, and only petals were collected, immediately frozen in liquid nitrogen, and stored at −80°C for further analysis. The flowers were divided into three stages according to color and size: S1, bud stage; S2, initial-flowering stage; and S3, full-flowering stage.

### Flower Color Measurement

The colors of fresh petals were measured and described according to the Royal Horticultural Society Color Chart with a CR-400 chroma meter (Konica Minolta, Tokyo, Japan). Three parameters, L^*^, a^*^, and b^*^ were determined: L^*^ (ranging from 0 to 100) indicates lightness. Positive and negative values of a^*^ standard for red and green, respectively, and those of b^*^ represent yellow and blue, respectively (McGuire, 1992; Liu et al., 2016). Five independent biological replicates were obtained to calculate the mean.

### Pigment Extraction and Analysis of Total Anthocyanin Content

To extract pigment, plant samples (0.5 g) were ground into powder in liquid nitrogen and added to 10 mL of methanol. The extraction was incubated for 48 h at 4°C away from light with vortexing every 8 h. The supernatant was removed after 10 min centrifugation (6,000 rpm) and stored at −20°C for further analysis. Three biological replicates were prepared for analysis.

The total anthocyanin (TA) content was determined according to literatures (Li et al., 2012) with a slight change. Hydrochloric acid (24 µL of 12 M) was added to 800 µL of the extraction, and the mixture was incubated for 15 min at room temperature. The absorbance of the reaction mixture was measured immediately at 530, 620, and 650 nm. The content of total anthocyanin was calculated using a standard curve of cyanidin-3-galactoside. The results are presented as milligrams per gram fresh weight (mg/g FW). Three biological replicates and three technical replicates were analyzed.

### HPLC-Diode Array Detector Analysis

The samples were filtered through 0.45 μm ﬁlters and the phenolic compounds were analyzed using HPLC coupled with DAD; HPLC-diode array detector (HPLC-DAD) analysis was carried out using a Shimadzu LC-2030C liquid chromatograph (Shimadzu, Kyoto, Japan) equipped with an Inertsil C-18 column (5.0 μm particle size, 4.6 mm × 250 mm). The HPLC-DAD separation was performed as previously described by Han et al. (2020). The compound varieties were determined by comparing their retention times and UV spectral data with those of the known standards according to reported LC-MC and NMR spectroscopic results of *Malus* spp. (Li et al., 2007; Hu et al., 2017). The concentration of each compound was calculated using corresponding standard calibration curves with three biological replicates.

### RNA Isolation and Library Construction for Transcriptomic Analysis

Total RNA was extracted from petals using a Trizol reagent kit (Invitrogen, Carlsbad, CA, USA), according to the manufacturer’s protocol. Three biological replicates were analyzed to conducted RNA sequencing experiments. The RNA quality was assessed using an Agilent 2100 bioanalyzer (Agilent Technologies, Palo Alto, CA, USA) and checked using RNase free agarose gel electrophoresis. After total RNA was extracted, eukaryotic mRNA was enriched using Oligo (dT) beads. The enriched mRNA was then fragmented into short fragments using fragmentation buffer and reverse transcripted into cDNA with random primers. Second-strand cDNA was synthesized by DNA polymerase I, RNase H, dNTP, and buffer. Then, the cDNA fragments were purified using a QiaQuick PCR extraction kit (Qiagen, Venlo, The Netherlands), end-repaired, poly(A) tails added, and ligated using Illumina sequencing adapters.

The RNA sequencing and assembly were performed using Illumina HiSeq2500 by Gene Denovo Biotechnology Co. (Guangzhou, China). Pair ends were used for the sequencing approach, and the read length was 150 nt. Clean reads were obtained by removing reads containing adapter and more than 10% of unknown nucleotides (N), as well as low-quality sequence containing more than 50% low-quality (*Q* ≤ 20) bases from raw data, and were aligned to *Malus × domestica* HFTH1 Whole Genome version 1.0 (https://www.rosaceae.org/species/malus_×_domestica_HFTH1/genome_v1.0) using Bowtie (Langmead and Salzberg, 2012) and HISAT2 (Kim et al., 2015). The “-RNA- strandness RF” was set to 2.4, and the other parameters were set as default. The mapped reads of each sample were assembled using StringTie v1.3.1 in a reference-based approach (reference sequence: *Malus* × *domestica* HFTH1). For each transcription region, an FPKM (fragment per kilobase of transcript per million mapped reads) value was calculated to quantify its expression abundance and variations using StringTie software.

### DEG Analysis

DEG analysis of three stages of flowers was performed using the DEGseq R package; *q* < 0.005, FDR<0.05, and |log2(foldchange (FC))| >1 were set as the thresholds for significantly differential expression. If a gene was only expressed in one treatment, then the expression in other treatments was calculated at the minimum value of 0.01. The gene function of DEGs was annotated according to the following databases: NCBI nonredundant protein sequences (Nr), NCBI nonredundant nucleotide sequences (Nt), Pfam (Protein family); clusters of orthologous groups of proteins (KOG/COG), Swiss-Protein (a manually annotated and reviewed protein sequence database), Kyoto Encyclopedia of Genes and Genomes (KEGG) Ortholog database (KO), and Gene Ontology (GO). GO includes three ontologies: molecular function, cellular component, and biological process. The basic unit of GO is the GO-term. First, all DEGs were mapped to GO terms in the GO database (http://www.geneontology.org/), gene numbers were calculated for every term, and significantly enriched GO terms in DEGs, compared with the genome background, were defined by hypergeometric testing. The KEGG is the major public pathway-related database. Pathway enrichment analysis identified significantly enriched metabolic pathways or signal transduction pathways in DEGs relative to the whole genome background.

### Homolog Search and Phylogenetic Tree Construction

A total of 32 MYB genes were isolated from DEGs of *M. halliana* and translated into protein using the NCBI Open Reading Frame Finder (http://www.ncbi.nlm.nih.gov/projects/gorf). Sequences of *Arabidopsis* AtMYB proteins were retrieved from the UniProt Database (http://www.uniprot.org). The evolutionary history was inferred using the neighbor-joining method (Saitou and Nei, 1987). The bootstrap consensus tree inferred from 1,000 replicates (Felsenstein, 1985) was taken to represent the evolutionary history of the taxa analyzed (Felsenstein, 1985). Branches corresponding to partitions reproduced in less than 50% bootstrap replicates were collapsed. The evolutionary distances were computed using the *p*-distance method (Nei and Kumar, 2000) and expressed in the units of the number of amino acid differences per site. All ambiguous positions were removed for each sequence pair. Evolutionary analyses were conducted in MEGA6 (Tamura et al., 2013).

### Quantitative Real-time PCR Analysis

Total RNA was isolated from the frozen sample following the method described previously. Approximately 1 µg of total RNA was used for first-strand cDNA synthesis using a PrimeScriptTM RT reagent kit with a gDNA Eraser (TaKaRa BioInc., Shiga, Japan) kit, following the manufacturer’s instructions. Quantitative RT-PCR was performed based on the 2 × plus SYBR real-time PCR mixture (Bioteke Corporation, Beijing, China). Samples for qRT-PCR were run in three biological replicates and three technical replicates; 18s RNA was used as the reference gene (Lu et al., 2017). The mean expression levels of the genes of interest were normalized to the relative expression level. Specific primers were designed from the selected gene sequences using Primer 5.0 and the primer sequences are given in **Supplementary Table S1**.

### DNA Isolation and *MhMYB10* Sequence Clone

Total DNA was extracted from petals using a DNA extraction kit (Bioteke, Beijing, China) according to the manufacturer’s protocol. Full-length coding region DNA and the 2,129 bp promoter sequence of *MhMYB10* were isolated from petals according to the EU518249.2 sequence in NCBI (https://www.ncbi.nlm.nih.gov/) and using primers listed in **Supplementary Table S1**. The PCR reactions were performed using TransStart Fastpfu DNA Polymerase, following the manufacturer’s instructions (Transgen Biotech, Beijing, China). The PCR products were purified following the manufacturer’s instructions (Bioteke Corporation, Beijing, China). The DNA fragments from three independent biological replicates were cloned using the pMD19-Teasy vector (Takara Bio Tech Co. Ltd., Beijing, China), and sequenced by Augct Co (Augct, Xi’an, China).

### Methylation Assay of *MhMYB10* Promoter

Bisulfite sequencing analysis was used to measure the methylation levels of the *MhMYB10* promoter as described by Telias et al. (2011), with three biological replicates. Briefly, 1 µg of gDNA was treated using the DNA bisulfite conversion kit (Tiangen Biotech Co. Ltd., Beijing, China). Using the treated DNA as a template, the targeted *MhMYB10* promoter fragments were amplified using 2 × Taq Master Mix (Novoprotein Co., Shanghai, China) with degenerate primers (**Supplementary Table S1**), ligated into the pMD19-T vector (Takara Bio Tech Co. LTD, Beijing, China), and then sequenced by Augct company (Augct, Xi’an, China). Sequences of 10 independent clones per biological replicate were obtained and analyzed using the Kismeth online software (Gruntman et al., 2008), and the methylation level of the targeted fragments was calculated based on the percentage of detected cytosines in methylated DNA sequence relative to the reference sequence.

## Results

### Analysis of Flower Phenotype and Phenolic Compounds

The petal color of *M. halliana* was significantly faded from red to pale pink during developmental stages from S1 to S3 (**Figure 1A**). Consistently, the chromatic parameters of petals showed that L^*^ values increased significantly, and a^*^ and b^*^ values decreased during flower development (**Figure 1B**). The total anthocyanin (TA) contents dropped from S1 to S3 (**Figure 1C**).

Furthermore, the HPLC-DAD results demonstrated that 13 types of phenolic compounds were detected in petals; these includes anthocyanins, flavonols, flavanols, flavones, and phloridzin. Of four kinds of cyanin glycoside detected (**Table 1**), cyanidin-3-galactoside was the major anthocyanin, and its concentrations were the highest compared with other anthocyanins, flavonols, flavanols, and flavones in petals. The highest concentration of cyanidin-3-galactoside (S1) was 3.4 times greater than the smallest (S3). In addition, the abundant phloridzin was determined in petals, and the concentration decreased from S1 to S3. The concentrations of other compounds remained low and stable during flower development.

**Table 1:** The HPLC-DAD results of petal extraction of *Malus halliana*.

Classification	Polyphenol (mg/g)	S1	S2	S3
Anthocyanin	Cyanidin-3-O-galactoside	1.752 ± 0.052^a^	1.075 ± 0.742^ab^	0.474 ± 0.107^b^
	Cyanidin-3,5-O-diglucoside	0.062 ± 0.001^a^	0.013 ± 0.001^b^	0.005 ± 0.001^c^
	Cyanidin-3-O-arabinoside	0.185 ± 0.0005^a^	0.175 ± 0.020^a^	0.160 ± 0.022^a^
	Cyanidin-3-O-rutinoside	0.368 ± 0.008^a^	0.382 ± 0.007^a^	0.421 ± 0.056^a^
Flavonol	Quercetin-3-O-glucuronide	0.632 ± 0.015^a^	0.535 ± 0.011^ab^	0.543 ± 0.076^b^
	Hyperoside	0.270 ± 0.005^a^	0.277 ± 0.004^a^	0.301 ± 0.039^a^
	Taxifolin	1.361 ± 0.012^a^	0.868 ± 0.040^b^	0.741 ± 0.124^b^
	Kaempferol-3-glucoside	0.192 ± 0.003^b^	0.194 ± 0.0002^b^	0.437 ± 0.079^a^
Flavanol	Catechin	0.022 ± 0.002^c^	0.045 ± 0.006^b^	0.032 ± 0.008^a^
	Epicatechin	0.104 ± 0.010^a^	H0.091 ± 0.011^ab^	0.069 ± 0.016^b^
Flavone	Naringenin	0.019 ± 0.002^b^	0.021 ± 0.006^b^	0.056 ± 0.001^a^
	Eriodictyol	0.278 ± 0.005	ND	ND
Dihydrochalcone	Phloridzin	34.819 ± 1.133^a^	20.648 ± 0.711^b^	7.952 ± 1.317^c^

The results are presented as mean ± SD. Different letters between cultivars denote significant differences (Duncan test, p < 0.05).

### Transcriptomic Assembly and Expression Analysis of DEGs

The RNA sequencing results of *M. halliana* petals showed that, of the total clean reads from the samples, 87.69–89.48% matched the *Malus* domestica genome (HFTH1 Whole Genome v1.0) with 84.85‒87.13% unique mapped reads and 2.53–2.84% multiple mapped reads (**Supplementary Table S2**). The total number of sequenced genes was 33,819, including 31,660 sequenced reference genes and 2,159 new genes (**Supplementary Table S3**).

**Figure 2:**
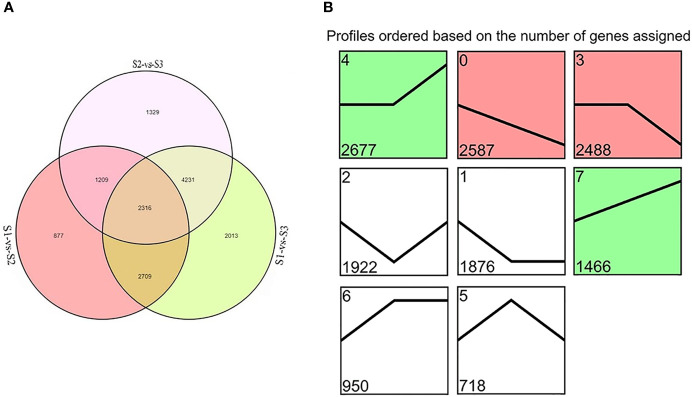
The differentially expressed genes (DEGs) screened by RNA-Seq analysis during flower developmental stages of *Malus halliana*. **(A)** Venn diagram of DEGs in three comparisons (S1-vs-S2, S1-vs-S3, and S2-vs-S3, respectively). **(B)** Trend analysis of DEG expression during flower developmental stage (from S1 to S3). Colored block trend: significant enrichment trend (Q ≤ 0.05). Green color indicated upregulation trend and red color indicated downregulation trend. Without color trend: the enrichment of trends.

DEGs were identified between samples of each treatment group. Totals of 7,111, 11,269, and 9,085 DEGs were detected in S1 vs. S2, S1 vs. S3, and S2 vs. S3, respectively. Overall, 2,316 genes were detected in all three comparisons (**Figure 2A**). The KEGG pathway enrichment analyses were used to identify the functions enriched in DEGs (**Supplementary Figure S1**). The most heavily enriched KEGG pathways were related to the metabolic pathway and biosynthesis of secondary metabolites. Of these pathways, the phenylpropanoid biosynthetic pathway included 77, 115, and 108 DEGs, and the flavonoid biosynthetic pathway included 33, 41, and 33 DEGs for S1 vs. S2, S1 vs. S3, and S2 vs. S3, respectively. These pathways are involved in anthocyanin biosynthesis, suggesting that these DEGs had important effects on flower color formation of *M. halliana*.

A trend analysis was carried out to explore the expression patterns of all DEGs in detail (**Figure 2B**). All DEGs were clustered into eight profiles, of which four trend profiles (0, 4, 3, and 7) showed high enrichment (*p* < 0.01) with the colored block, and four profiles represented the enrichment of significant trends without color. The expression of 2,587 genes displayed a reducing trend during the whole flower development in Profile 0, and the expression of 1,466 genes demonstrated an opposite trend in Profile 7. The expression of 2,677 genes showed no difference in S1 vs. S2, but subsequent extreme reduction in S2 vs. S3 in Profile 3. However, the expression of 2,488 genes exhibited no change in S1 vs. S2 but subsequent increased in S2 vs. S3 in Profile 4. Based on the pattern of the flower color fading, we focused on Profile 0 and Profile 7 in the subsequent analyses.

### Dynamic Changes of DEGs are Related to Anthocyanin Metabolism

**Figure 3:**
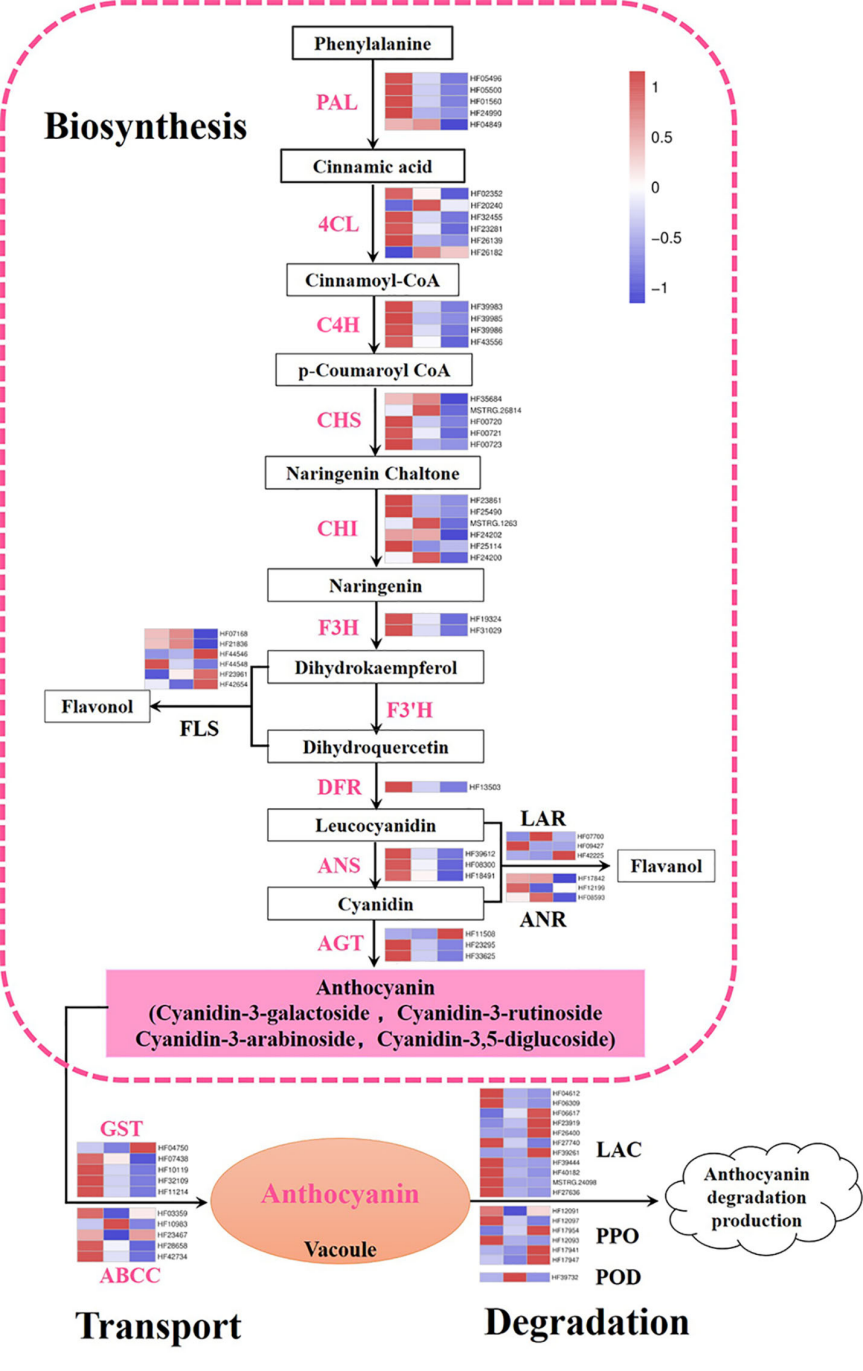
Expression pattern of genes involved in anthocyanin biosynthetic, degradation, and transport pathway. Color boxes from left to right represent genes expression level at S1, S2 and S3. PAL, phenylalanine ammonia lyase; 4CL, 4-coumarate coenzyme A ligase; C4H, cinnamate 4-hydroxylase; CHS, chalcone synthase; CHI, chalcone isomerase; F3H, flavanone 3-hydroxylase; F3’H: flavonoid 3’-monooxygenase; DFR, dihydroflavonol-4-reductase; ANS, anthocyanidin synthase; AGT, anthocyanidin 3-O-glucosyltransferase; FLS, flavonol synthase; LAR, leucoanthocyanidin reductase; ANR, anthocyanidin reductase; GST, glutathione S-transferase; ABCC, ATP-binding cassette; LAC, laccase; PPO, polyphenol oxidases; POD, class III peroxidases. Color saturation represents the normalize expression level of genes at S2 and S3 with S1 based on fold change, the color gradient on the right, ranging from blue to white to red represents low, middle, and high values of gene expression.

In this work, genes participating in the anthocyanin metabolic pathway were screened and expression heat maps were constructed based on FPKM (**Table S4**; **Figure 3**). A total of 47 genes were involved in anthocyanin biosynthesis. The predicted proteins encoded by upstream genes included five PAL, six 4CL, four C4H, five CHS, six CHI, and two F3H. The predicted proteins encoded by downstream genes contained one DFR, three ANS, and three anthocyanidin 3-O-glucosyltransferase (AGT). Almost all genes previously described were significantly downregulated during flower fading, suggesting that they play important roles in anthocyanin biosynthesis. In particular, *MhPAL* (HF01560), *MhCHS* (HF00720 and HF00721), *MhCHI* (HF23861 and HF25490), *MhDFR* (HF13503), and *MhANS* (HF39612 and HF08300) had especially high expression levels at S1, which decreased dramatically along with the fading of flower color. These genes were considered the key candidate genes affecting the anthocyanin biosynthesis of *M. halliana* flowers. Some branch genes in the anthocyanin biosynthetic pathway were also studied, including six flavonol synthases (*FLS*), three leucoanthocyanidin reductases (*LAR*), and three anthocyanidin reductase (*ANR*) genes, which did not show a consistent expression pattern.

In addition, 10 predicted coding genes involved in anthocyanin transport were identified, including five *GST*s and five *ABCC*s. The expression levels of two *MhGST* genes (HF07438 and HF32109) were very high and the differences at different stages were highly significant. Moreover, we analyzed the anthocyanin degradation-related genes, including *POD*, *PPO*, and *Lac* genes. Most of the degradation genes were expressed at low levels, except HF17954 (*MhPPO*) whose expressions increased significantly as flower color fading (**Figure 3**).

### Expression Profiling of Transcription Factors Associated with Anthocyanin Biosynthesis

In this study, to identify the putative TFs that regulate anthocyanin biosynthesis, all MYBs (MYB domain proteins), bHLHs (basic helix-loop-helix), WD40s, ERFs (ethylene-responsive transcription), bZIPs (basic region-leucine zipper), NACs (NAM/ATAF/CUC), WRKYs (WRKY proteins), and MADS-boxes (AGL and SOC) were selected and analyzed in detail in DEGs (**Table 2**). Of these TFs, MYBs were the largest family with 111 genes, followed by NACs (94), ERF (88), and bHLH (79), while WD40s, MADS-boxes, and bZIPs had fewer genes. The expression heat maps of these TFs were constructed based on FPKM, and the complex expression patterns were present in TFs (**Supplementary Figure S3**). Further, we also screened TFs belonging to Profile 0 and Profile 7 that were consistent with and the opposite to the changing pattern of flower color, respectively. There were 27 MYBs in Profile 0 (such as HF36879, HF02692, and HF33298) and only five in Profile 7 (such as HF38722, and HF17885), indicating that most MYBs activated anthocyanin biosynthesis. Similarly, 18 bHLHs, five WD40s, nine ERFs, and three MADS-boxes were identified in Profile 0, while eight bHLHs, four ERFs, and one WD40 were identified in Profile 7. However, more NACs, WRKYs, and bZIPs were present in Profile 7 than those in Profile 0 (**Table 2** and **Supplementary Figure S3**).

**Table 2:** Candidate anthocyanin regulatory genes in *Malus halliana*.

Gene family	NO. all^a^	S1 vs. S2		S1 vs. S3		S2 vs. S3		S1 vs. S2 vs. S3	
		NO. up^b^	NO. down^c^	NO. up	NO. down	NO. up	NO. down	Profle 0	Profile 7
*MYB*	111	16	53	31	61	29	31	27	5
*NAC*	94	18	34	47	32	44	17	10	19
*ERF*	88	13	36	24	25	40	18	9	4
*bHLH*	79	9	35	26	36	41	19	18	8
*WRKY*	57	6	19	39	6	43	2	1	8
*WD40*	25	4	12	4	13	4	4	5	1
*MADS-box*	23	3	4	5	15	8	9	3	0
*bZIP*	20	7	1	6	10	2	12	0	3
Total	497	76	194	182	198	211	112	73	48

^a^indicates the total number of regulatory genes in DEGs, ^b^indicates the number of upregulated genes in each comparison, and ^c^indicates the number of downregulated genes in each comparison. All candidate anthocyanin regulatory genes in this table are screened from DEGs.

### MYB Family Phylogenetic Tree Construction

**Figure 4:**
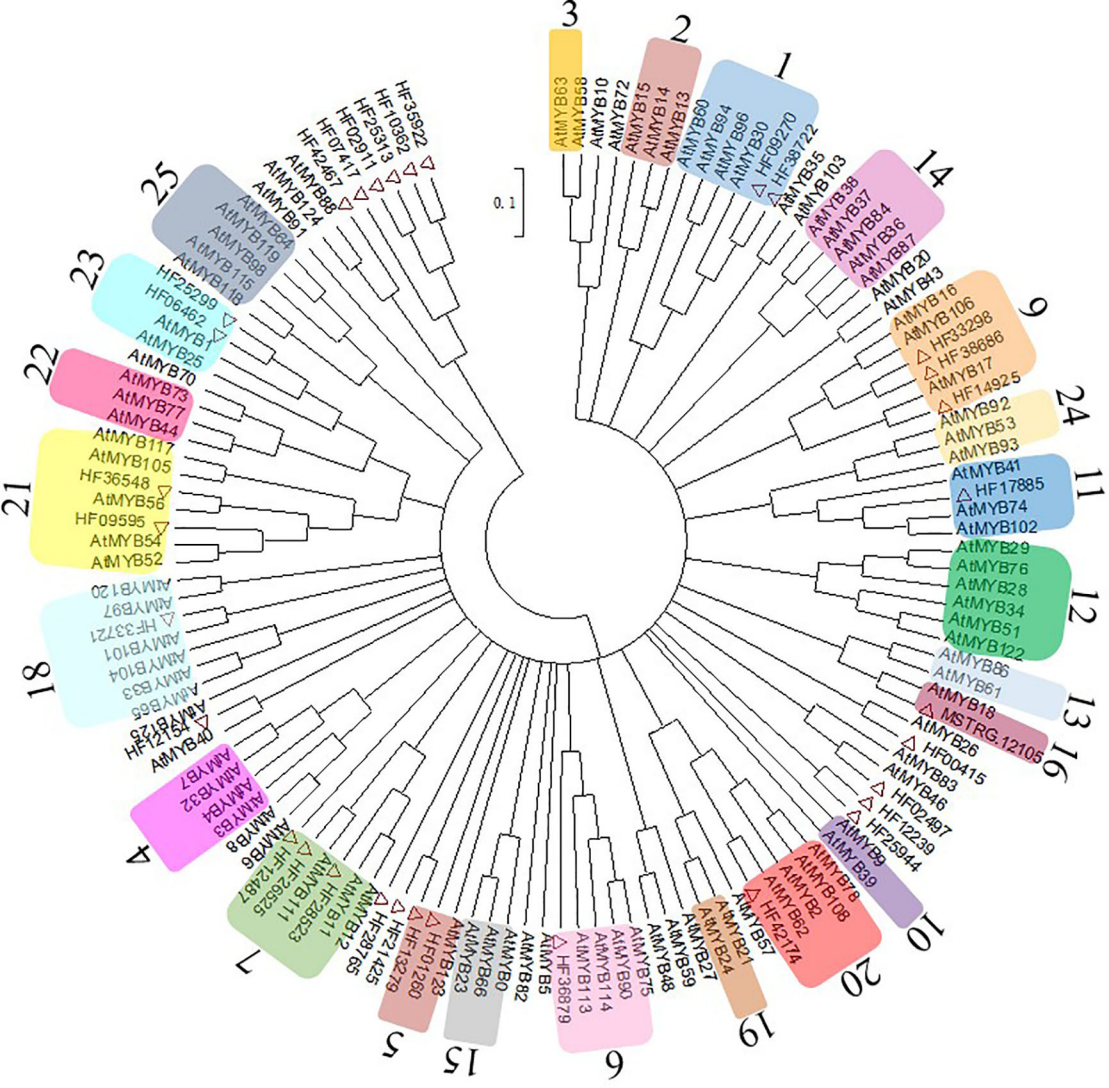
Evolutionary relationships of *MhMYBs*. Full-length amino acid sequences of R2R3-MYBs from the chrysanthemum transcriptome dataset and arabidopsis genome were first aligned using ClustalW in MEGA6. The phylogenetic tree was constructed according to the neighbor-joining method. Branches corresponding to partitions reproduced in less than 50% of the bootstrap replicates were collapsed. The evolutionary distances were computed using the p-distance method. Thirty-two MhMYBs marked red triangle were identified from the RNA sequencing data of *M. halliana*. The arabic numerals on the cycle indicated gene families.

The MYBs are the largest TF family related to anthocyanin biosynthesis. In this study, 32 MYBs were identified from RNA sequencing data and 99 *Arabidopsis* MYBs from the UniProt Database (http://www.uniprot.org) were used to conduct a phylogenetic tree (**Figure 4**). The MYBs were integrated with AtMYBs in clustered phylogenetic clades or subclades and divided into 23 MYB subgroups based on the available literature (Stracke et al., 2001), and there were no genes in Sg8 and Sg17. The MYBs in Sg6 were reported to be regulators related to anthocyanin biosynthesis (Stracke et al., 2001). In the present study, only one MYB (HF36879, renamed *MhMYB10*) belonged to Sg6.

### Expression Profile of the Selected Genes in Diverse *Malus* Spp.

**Figure 5:**
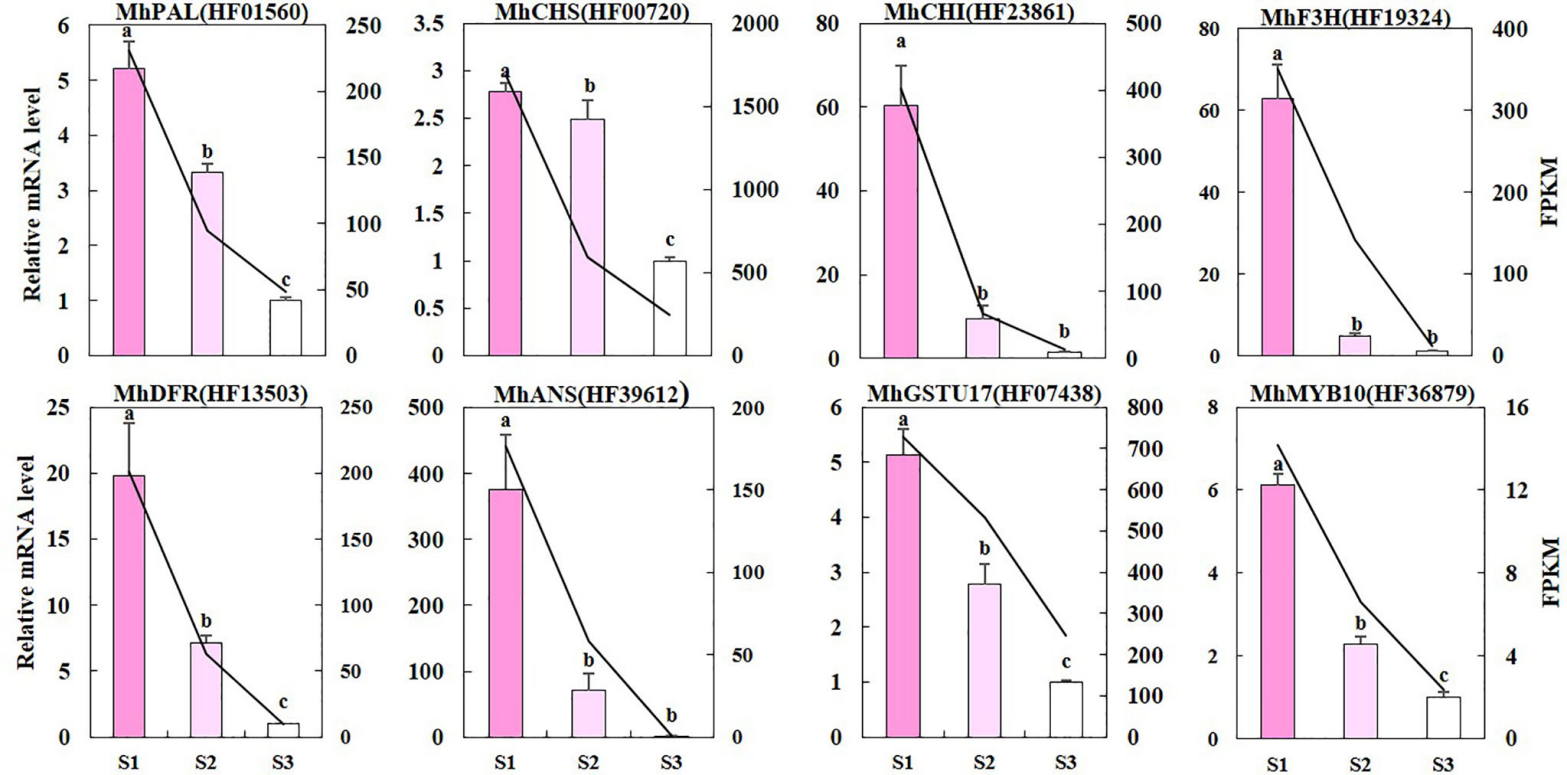
The qRT-PCR validation of DEGs. The left y-axis denotes the RNA relative expression obtained by qRT-PCR. Error bars indicate the standard errors. Different letters between cultivars denote significant differences (Duncan test, *p* < 0.05). The right y-axis represents the fragments per kilobase per million fragments (FPKM) value of each gene using RNA sequencing analysis.

We used qRT-PCR to validate whether the difference in RNA sequencing levels of DEGs in the anthocyanin biosynthetic pathway truly reflects the actual transcription level (**Figure 5**). The expression patterns of nine genes (*MhPAL* (HF01560), *MhCHS* (HF00720), *MhCHI* (HF23861), *MhFHT* (HF19324), *MhDFR* (HF13503), *MhANS* (HF39612), *MhFLS* (HF44548), *MhGSTU17* (HF07438), and *MhMYB10* (HF36879) obtained by qRT-PCR were consistent with the RNA sequencing data, confirming the validity of our results. It is worth noting that the reduction in most of these gene expressions was greater from S1 to S2 than that from S2 to S3.

**Figure 6:**
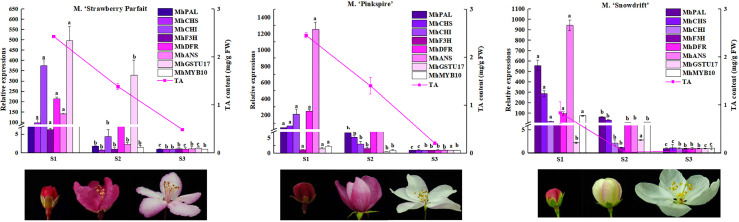
Anthocyanin contents and the key DEGs transcript levels in anthocyanin metabolism in three *Malus* flower in varying colors. The relative expressions of genes are exhibited as column diagram. Anthocyanin levels are indicated by lines. Correlation coefficient values between gene expression level and anthocyanin content are presented above each gene legend correspondingly. Different letters between cultivars denote significant differences (Duncan test, *p* < 0.05).

Meanwhile, these gene expression levels were analyzed in other *Malus* spp. with varying flower phenotypes to confirm their wide function in flower color formation (**Figure 6**). The flower of *M*. ‘Strawberry Parfait’ is rose at S1 and fades to light-pink at the flower opening, and *M*. ‘Pinkspires’ has a pink flower at S1 and fades to white at S3. The flowers of *M*. ‘Snowdrift’ are pink at S1 and quickly change to white at S2. Consistent with flower color characteristics, TA in petals declined dramatically during flower development; at the same time, *M*. ‘Strawberry Parfait’ and *M*. ‘Pinkspires’ had higher TA than *M*. ‘Snowdrift’.

Of the nine genes selected, the expressions of *MhPAL*, *MhCHI*, *MhDFR*, and *MhANS* decreased remarkably in three *Malus* spp. during flower fading. Correlation analysis showed an extremely significant correlation between the expressions of *MhPAL*, *MhCHS*, *MhCHI*, *MhDFR*, *MhANS*, *MhMYB10*, and TA contents in all three *Malus* spp. For *M*. ‘Strawberry Parfait’ and *M*. ‘Snowdrift’, *MhF3H* expression was closely related to TA content.

### Methylation of *MhMYB10* Promoter at Flower Fading

**Figure 7:**
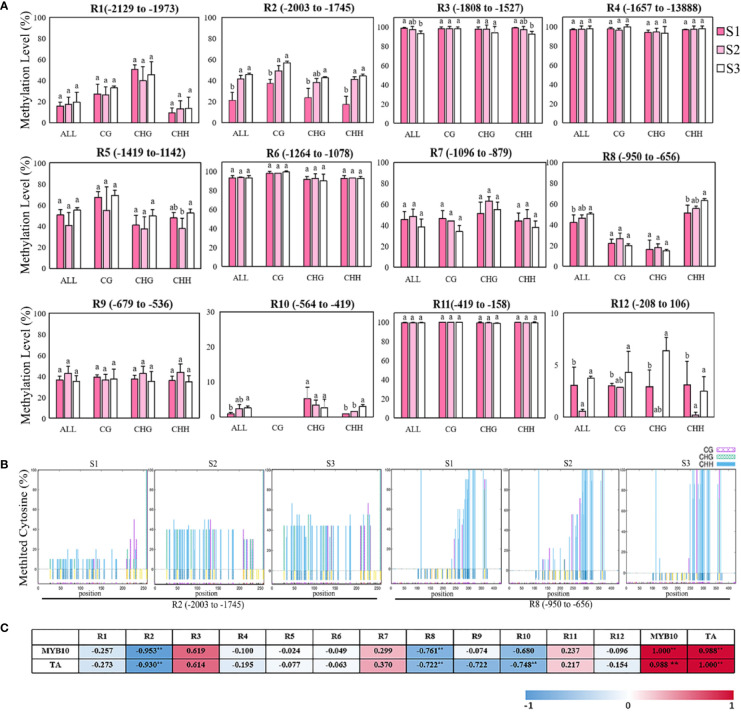
Bisulfite sequencing analysis of cytosine methylation levels of different regions in *MhMYB10* promoter at the different flower stages. **(A)** The methylation levels of *MhMYB10* promoter at the different stages. Each data point represents a mean ± SD of three independent DNA extractions with three independent technical replicates. Ten independent clones from each reaction were sequenced and analyzed. In the x axis, “All” refers to overall methylated cytosines, while CG, CHG and CHH refer to the three different contexts of cytosines, in which H represents nucleotide A, C, or T. Different letters between cultivars denote significant differences (Duncan test, *p* < 0.05). **(B)** The methylation levels of different cytosine contexts (CG, CHG, CHH, H represents nucleotide A, C, or T) in R2 and R8 regions at the different stages. **(C)** The correlation analysis between the methylation level of *MhMYB10* and *MhMYB10* expression and anthocyanin content.

The previous results show that the repression of *MhMYB10* expression probably plays a key role in the flower color fading of *Malus* spp. during development. To examine whether there is an alteration in the dynamics of methylation in the *MhMYB10* promoter during flower fading, 2,129 bp upstream sequences from the translation initiation site were isolated (**Supplementary Figure S2**) and the methylation levels of 12 regions were analyzed using bisulfite sequencing (**Figure 7A**). The highest methylation activities of *MhMYB10* occurred in R3 (−1,808 to −1,527 bp), R4 (−1,657 to −1,388 bp), R6 (−1,264 to −1,078 bp), and R11 (−419 to −158 bp) with more than 90% in all cytosine contexts (CG, CHG, and CHH) at three stages. The methylation level of R3 decreased somewhat during flower development. The methylation levels of R5 (−1,419 to −1,973 bp), R7 (−1,096 to −879 bp), and R9 (−679 to −536 bp) did not significantly differ during flower development. Although the methylation levels of R10 (−546 to −419 bp) and R12 (−208 to 106 bp) were low during flower opening, the methylation activity of R10 increased significantly and was detected only in CHG and CHH context.

Interestingly, the methylation activities in R1 (−2,129 to −1,973 bp), R2 (−2,003 to −1,745 bp) and R8 (−1,419 to −1,142 bp) visibly increased during flower opening. The total methylation levels were 21.25%, 41.67%, and 45.64% for R2, and 39.38%, 46.25%, and 50.14% for R8 at S1, S2, and S3, respectively. The methylation activity at S1 was less (24.39%) than that at S3 (**Figure 7A**). The methylation activity in R2 and R8 could be detected in all three cytosine contexts. By contrast, the highest methylation activity of R2 occurred in the CG context, but R8 in the CHH context was much higher than that in the CG and CHG contexts (**Figure 7B**).

The correlation analysis results showed that *MhMYB10* expression and TA content were significantly negatively related to the methylation level of R2 and R8 among 12 regions. The correlations between the R2 methylation level with *MhMYB10* expression and TA content were 0.953 and 0.930 (*p* < 0.01), respectively (**Figure 7C**).

## Discussion

### Anthocyanin Biosynthesis and Degradation Control Flower Color

It is a common phenomenon that the flower color often changes during development, acting as a signal for pollinators. In most cases, color changes during flower development are due to the induction of anthocyanin biosynthesis (Oren-Shamir, 2009). In this study, the concentration of cyanidin-3-O-galactoside, which is the most important anthocyanin in the *M. halliana* flower, decreased dramatically along with flower color fading, which is the main reason for floral color fading. In addition, other pigments, such as flavonols and flavanols, may act co-pigmentations of anthocyanin to participate in the flower color formation (Iwashina, 2015).

The decline of anthocyanin content is the result of the complex mechanics involved in anthocyanin biosynthesis, transport, and degradation (Oren-Shamir, 2009). In this study, DEG analysis results showed that 47 candidate genes were involved in anthocyanin biosynthesis, and the expression of almost all of these genes (especially *MhPAL*, *MhCHS*, *MhCHI*, *MhDFR*, *MhANS*) were downregulated during flower fading of *Malus* spp., and positively correlated to anthocyanin content. The repressions of these genes were closely related to the color fading of the flower. In addition, the expression levels of *MhGSTU17* and *MhGSTF12* were extremely high at S1 and decreased sharply after S1, suggesting that these two genes could be key players in anthocyanin transport. One *MhPPO* gene (HF17954) was upregulated as flower color faded, indicating that HF17954 might play a primary function in anthocyanin degradation of *M. halliana*. Wang et al. (2017) reported that three families (GST, ABCC, and MATE) involved in anthocyanin transport and that two families (Lac and POD) involved in degradation were identified in transcriptome analysis of pear color fading, which is different from *M. halliana*, whose *MhPPO* was responsible for anthocyanin degradation. Moreover, the flower development process of *M. halliana* occurs during a period of increasing temperature (from late March to late April in Shannxi Chinese). Anthocyanin biosynthesis is repressed, and catabolism is activated by high temperature (Oren-Shamir, 2009). Therefore, a change in environmental conditions may result in a decrease of anthocycanin content to promote flower fading of *M. halliana*.

Considering that a greater number of genes and higher expression intensities were identified in anthocyanin biosynthesis than those in transport and degradation during flower development, similar results were reported by Huang et al. (2020) for a study on the flower color variation of *Malus* spp., suggesting that the expression repression of anthocyanin biosynthetic genes is the major reason for the decline of anthocyanin content during flower development.

### Transcription Factors Involved in the Anthocyanin Biosynthetic Pathway

The anthocyanin biosynthetic pathway is regulated by the highly conserved MYB (Xu et al., 2015). In our study, MYBs were identified as the largest TF family, containing 111 genes. Among these MYBs, the expressions profiles of 27 genes were consistent with the changing trend of flower color, and only five genes had an opposite expression trend. These results indicated that more MYBs could promote anthocyanin biosynthesis. Furthermore, phylogenetic tree analysis revealed that, of 32 genes, only *MhMYB10* (HF36879) was clustered in Sg6, in which MYBs in *Arabidopsis* have been proven to directly regulate the anthocyanin biosynthetic pathway (Stracke et al., 2001). Recently, Huang et al. (2020) found, by transcriptome sequencing of different flower color *Malus* spp., that *MYB113*-like (HF36879, namely, *MYB10*) expressions in red petals were several thousand times greater than those in white flowers. Moreover, *MhMYB10* expression significantly reduced during color fading, and was positively correlated with anthocyanin content not only in *M. halliana* but also in other *Malus* spp. These results strongly suggested that *MhMYB10* played important roles not only in flower color variation but also in flower color fading of *Malus* spp. Furthermore, *MdMYB10* is thought to play an important role in the anthocyanin biosynthetic pathway of *M. domestica* by regulating *DFR* and *ANS* expression (Espley et al., 2007). Taken together, this evidence demonstrates that *MhMYB10* expression is downregulated during color fading to induce decreases in anthocyanin content.

In addition, many authors have reported other TFs that participate in anthocyanin biosynthesis. For example, when *Brassica oleracea NAC019* is overexpressed in *Arabidopsis*, anthocyanin content decreased (Wang et al., 2018), while *AtERF4* and *AtERF8* are associated with changes in the transcript levels of anthocyanin biosynthetic genes and mediate the production of anthocyanin biosynthetic genes (Koyama and Sato, 2018). When *Brassica napus WRKY41-1* is overexpressed in *Arabidopsis*, there is a significant increase in anthocyanin content (Duan et al., 2018). In the present study, a number of transcription factors were screened as candidates for participation in regulating the anthocyanin biosynthetic pathway, including NACs, ERFs, WRKYs, MADS-boxes, and bZIPs, with 28 found to be downregulated and 35 upregulated as the flower color faded. These results suggest that, unlike the function of the MBW complex, most of these families likely act as transcription repressors in anthocyanin biosynthesis to promote color fading, especially NAC and WRKY families. These results suggest directions for further study on the flower coloration of *Malus* spp.

### Epigenetic Regulation of *MhMYB10* Play an Important Role in Flower Color Fading

DNA methylation, a conserved epigenetic modification, plays an important role during plant growth and development, such as the processes of tomato and orange fruit ripening and coloration (Lang et al., 2017; Huang et al., 2019). Methylation in the promoter can directly inhibit gene transcription and expression (Zhang et al., 2018). Deng et al. (2015) reported on the methylation level of the *ANS* promoter in two *Nelumbo nucifera* cultivars resulting in different color phenotypes. In the present study, the methylation analyses results demonstrate that, of 12 regions in the *MhMYB10* promoter, the methylation activity of R2 (−2,003 to −1,745 bp) and R8 (−950 to −656 bp) significantly increased along with petal color fading in *M. halliana*, and was negatively related to the TA content and *MhMYB10* expression. These results suggest that the increasing methylation level of R2 and R8 could repress *MhMYB10* expression, thereby slowing the anthocyanin accumulation.

How the methylation level of the *MYB10* promoter affects fruit pigment has also been studied. Research on red and green striped apple skin showed that higher methylation activity of the *MdMYB10* promoter occurred in the region from −1,400 to −651 bp (Telias et al., 2011); moreover, the methylation levels of the fragments (−604 to −911 bp and −1,218 to −1,649 bp) in *PcMYB10* promoter were much greater in green pear skin than those in red pear skin (Wang et al., 2013). In the present study, however, high methylation activities (>90%) were detected in R3 (-1,808 to −1,527 bp), R4 (−1,657 to −1,388 bp), R6 (−1,264 to −1,078 bp), and R11 (−419 to −158 bp), and no significant differences were detected at the different stages. However, significant differences were observed in R2 (−2,003 to −1,745 bp) and R8 (−950 to −656 bp) during flower fading, which were distinct from the methylation function location during fruit coloration. The methylation activities of these two fragments were tissue-specific and might play important roles in the regulation of the *MhMYB10* promoter in flowers. The methylation level of R2 increased sharply from S1 to S2 (20.42%), consistent with the expression of *MhMYB10* and anthocyanin biosynthetic structure genes, demonstrating that the period from S1 to S2 is important for flower fading.

In all three cytosine contexts, the highest methylation levels of R2 were in CG, followed by CHG and CHH. Meanwhile, the methylation levels of all three cytosine contexts increased with flower fading. However, for R8, much greater methylation levels were detected in CHH than those in CG and CHG. A consistent trend with flower color fading was exhibited in CHH, but not in CG and CHG, indicating that hypermethylation of the CHH context in R8 has an important effect on *MhMYB10* expression. Similar results were found in other fragments from −2,585 to −2,117 bp of *MdMYB10* promoter of apple (El-Sharkawy et al., 2015) and −1,218 to −1,649 bp of *PcMYB10* of pear (Wang et al., 2013). However, in maize, DNA methylation related to color formation largely occurred in CG and CHG contexts (Sekhon and Chopra, 2009). This evidence reveals that ligneous plants might have a more complex methylation regulation mechanism.

The petal color fading of *M. halliana* is accompanied by aging of the flower. The natural process of flower senescence, broadly the combination of events that lead to the death of cells, tissues, or organs, can regulate flower color formation, gene expressions and DNA methylation (Teixeira da Silva et al., 2014; Zhang et al., 2018). In this study, most anthocyanin metabolism gene expression was regulated significantly, and methylation activities of R2 and R8 were gradually increased with flower development. During apple maturation, the methylation in the promoter of *MdMYB10* is developmentally controlled (El-Sharkawy et al., 2015). These results suggest that gene expression and *MhMYB10* methylation are regulated at the genetic and development levels.

## Conclusion

**Figure 8:**
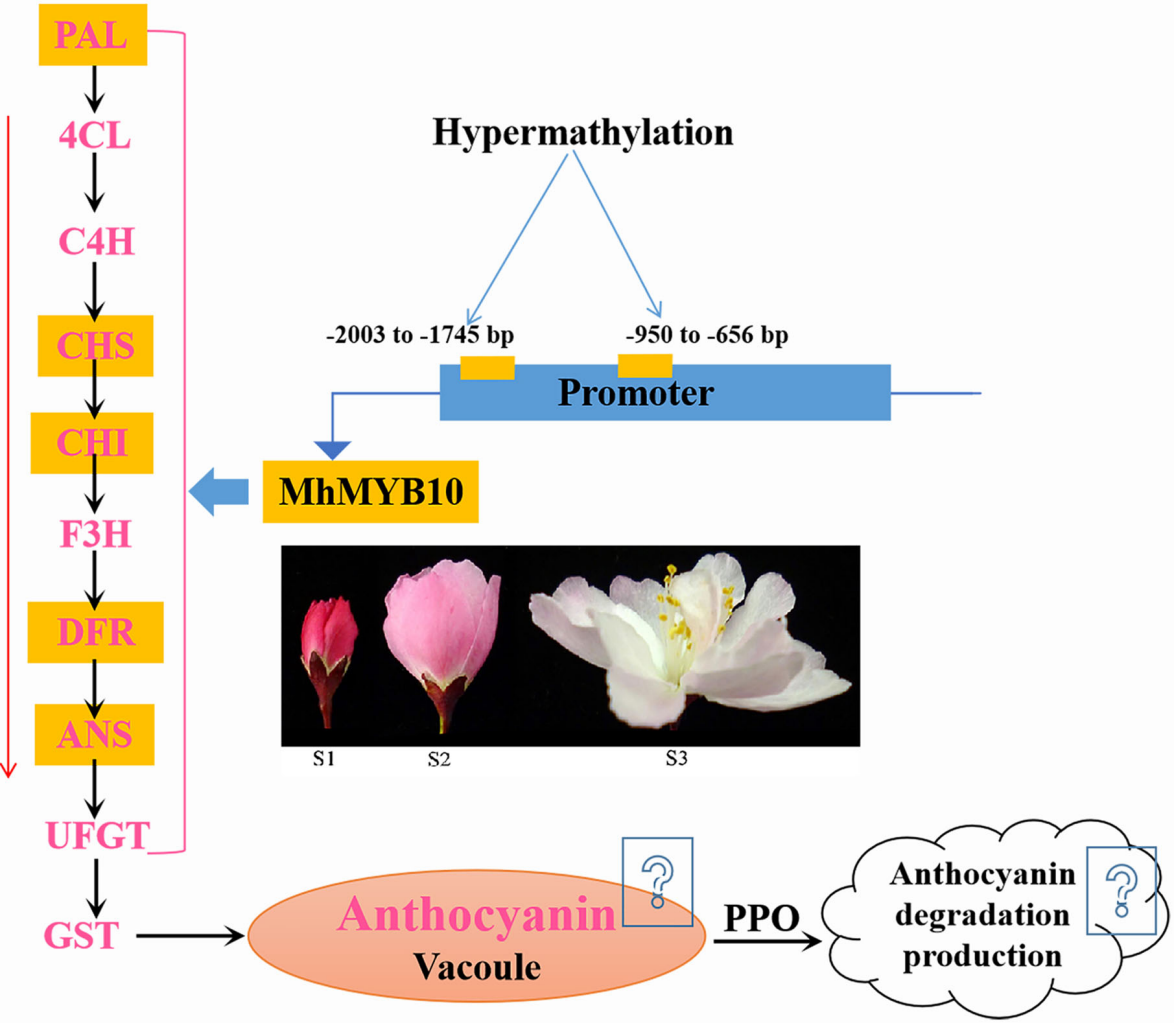
A simplified model of flower color fading of *M. halliana*. During flower development, hypermethylation in the *MhMYB10* promoter, especially −2003 to −1745 bp and −950 to −656 bp, repressed the expression of *MhMYB10*. *MhMYB10* influenced the transcription of structural genes (e.g., *MhANS*, *MhDFR*, and *MhCHS*), which decreased the anthocyanin biosynthesis. In additionally, genes expressions in anthocyanin transport and degradation were downregulated or upregulated, which may influence anthocyanin content. Finally, these complex process resulted in decline of anthocyanin content, so the flower color faded.

Through complete transcriptomic analysis of petals at the different development stages, we describe how anthocyanin biosynthetic, transport, and degradation pathway control flower color fading in *M. halliana* (**Figure 8**). Multiple genes were identified as the key genes that lead to a decrease of anthocyanin content, including *MhPAL*, *MhCHS*, *MhCHI*, *MhF3H*, *MhDFR*, *MhANS*, *MhGSTU17*, and *MhPPO*. Many transcription factors were down or upregulated during flower development, including MYBs, bHLHs, WD40s, WRKYs, NACa, ERFs, bIZPs, and MADS-boxes. The phylogenetic tree analysis found that, of the five MYBs that regulate anthocyanin biosynthesis, *MhMYB10* is a key transcription factor. Therefore, we next analyzed the methylation activity of the *MhMYB10* promoter. Methylation levels of R2 (−2,003 to −1,745 bp) and R8 (−950 to −656 bp) of the *MhMYB10* promoter increased as the flower color faded, suggesting that methylation of the *MhMYB10* promoter might play a critical role in the downregulation of anthocyanin biosynthetic genes, resulting in decreasing anthocyanin content and color fading.

## Data Availability Statement

The datasets generated for this study can be found in the NCBI Sequence Read Archive (SRA) with bioproject No. PRJNA658719, and under GenBank accession numbers of SAMN15887511, SAMN15887512, SAMN15887513, SAMN15887514, 538 SAMN15887515, SAMN15887516, SAMN15887517, SAMN15887518, 539 SAMN15887519.

## Author Contributions

M-LH and JY performed most of the experiments and data analysis. Y-HZ, X-WS, J-XM, and FZ carried out material collection and pigment extraction. Y-HZ and X-WS conducted pigment analysis. J-XM conducted a part of RNA extraction. M-LH, JY, and JZ participated in the preparation of the manuscript. H-HL conceived, designed and coordinated the studies. All authors contributed to the article and approved the submitted version.

## Funding

This work was supported by Shaanxi Academy of Forestry Science and Technology innovation plan special (SXLk2020-02-01) and National Natural Science Foundation of China (31570697).

## Conflict of Interest

Author FZ was employed by the company Shaanxi Qincao Ecological Environment Technology Co., Ltd.

The remaining authors declare that the research was conducted in the absence of any commercial or financial relationships that could be construed as a potential conflict of interest.
