# Supplementary material for: How the Color Fades From Malus halliana Flowers: Transcriptome Sequencing and DNA Methylation Analysis
Source: Front Plant Sci. 2020 Sep 23;11:576054. doi: 10.3389/fpls.2020.576054 (PMC7539061; doi:10.3389/fpls.2020.576054)
Supplement: Supplementary file 1 [file DataSheet_1.doc]

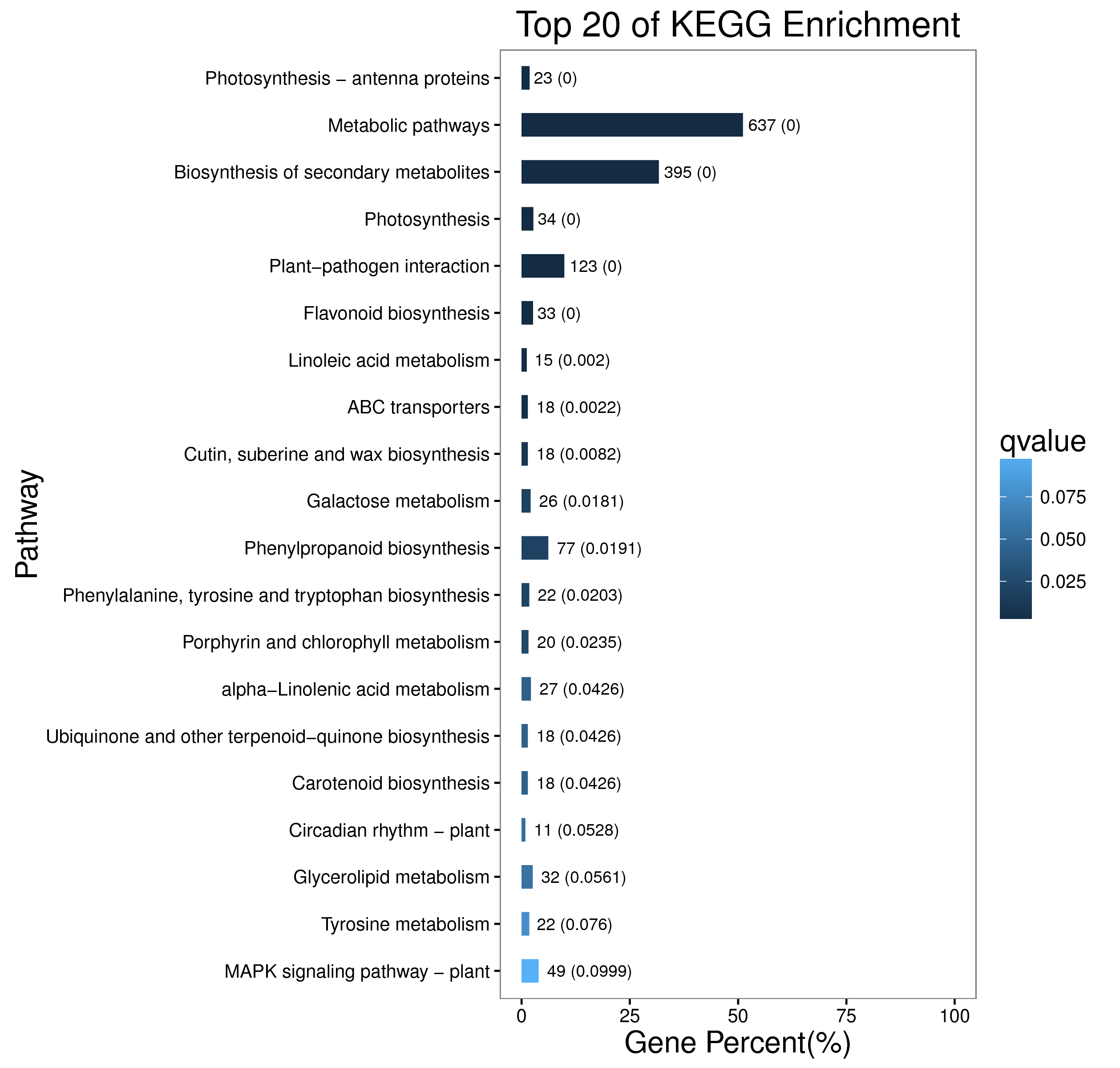


[
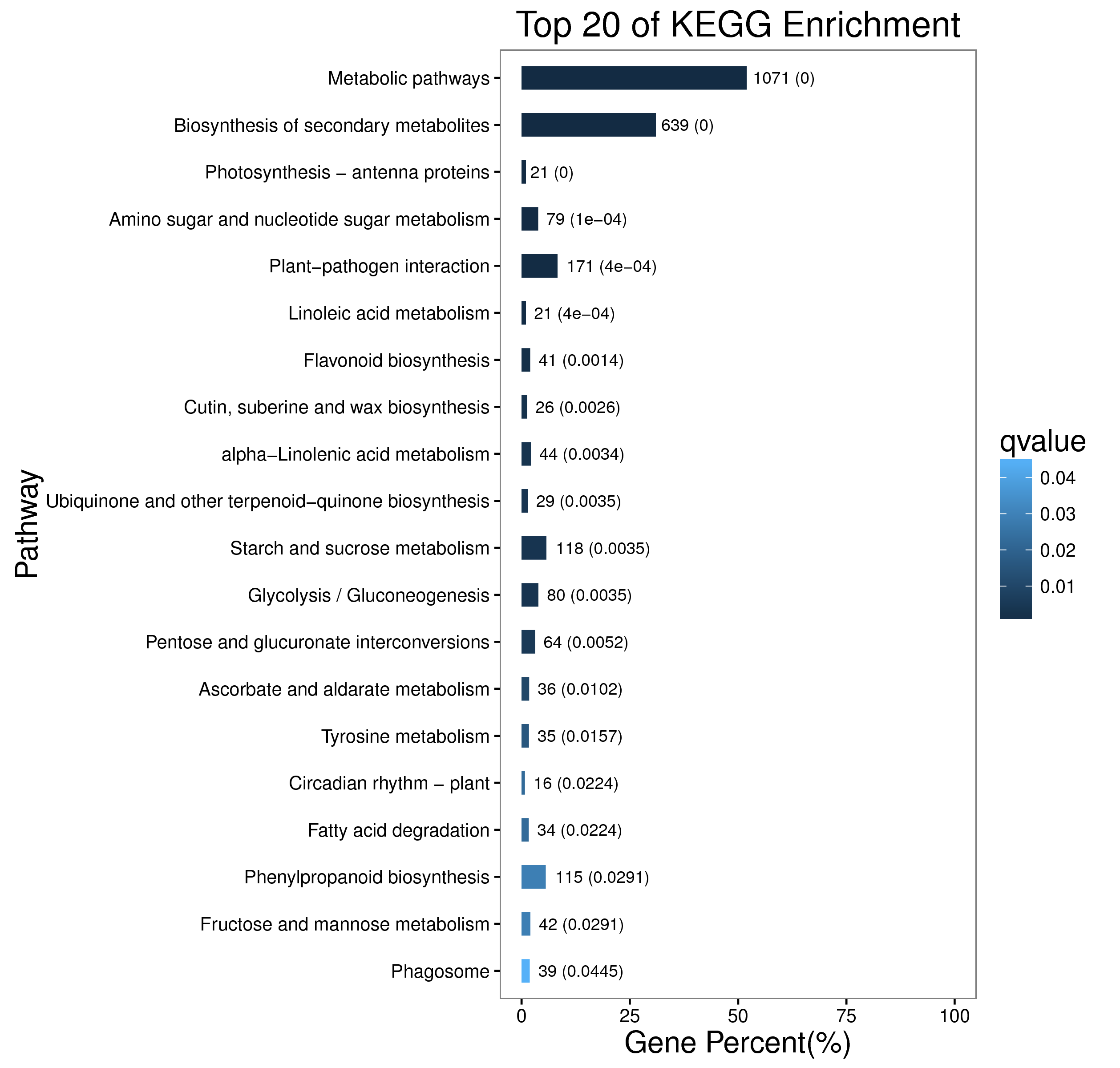
](file:///G://papers//我的//论文//myself//2019转录组//myself//Malus_halliana//GDR5827-Malus_halliana-result//7.GroupDiffExpression//enrich//KO//S1-vs-S3.barplot.png)

[
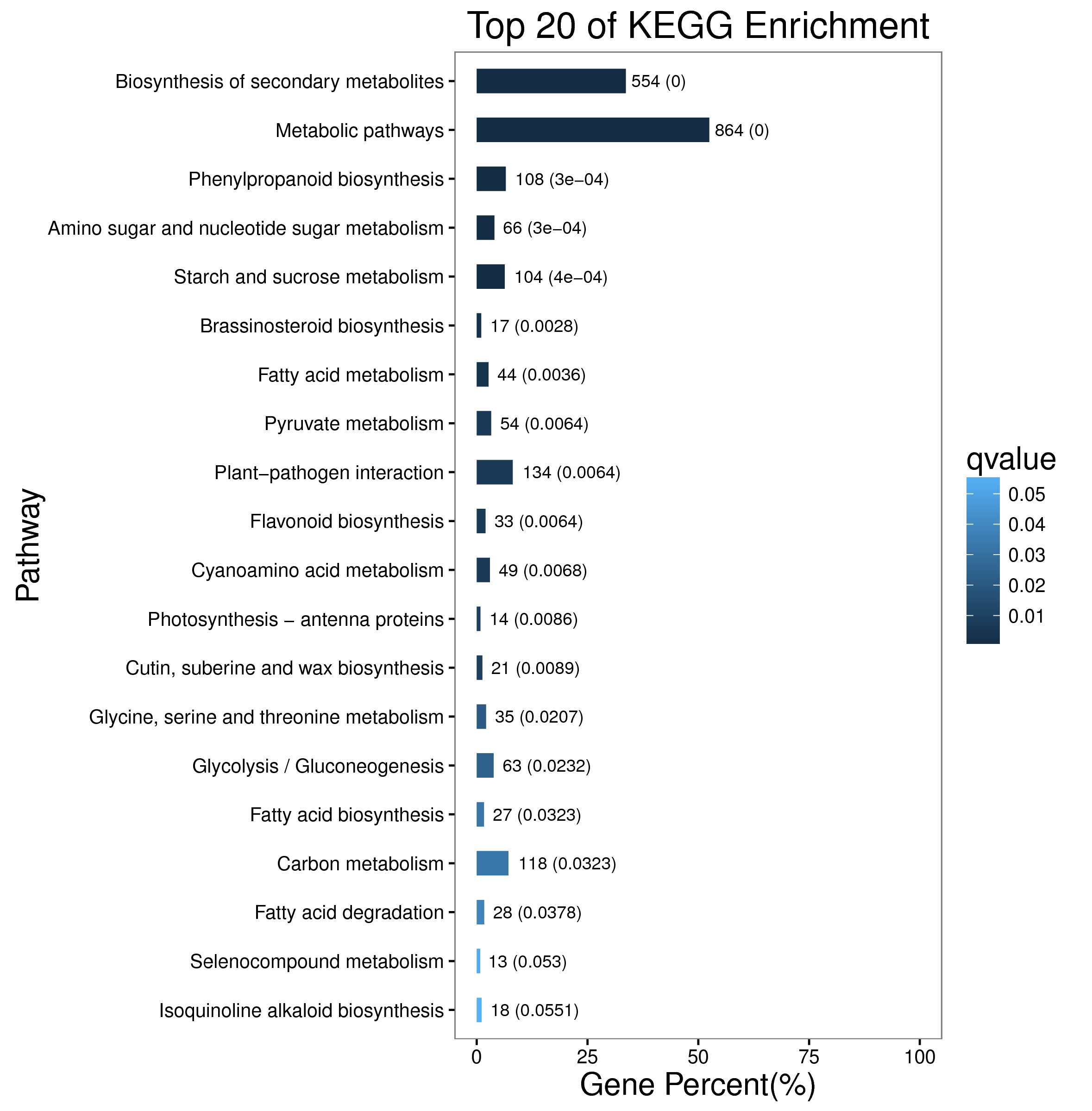
](file:///G://papers//我的//论文//myself//2019转录组//myself//Malus_halliana//GDR5827-Malus_halliana-result//7.GroupDiffExpression//enrich//KO//S2-vs-S3.barplot.png)

**Supplementary Figure 1.** KEGG pathway enrichment of DEGs
