# Supplementary material for: How the Color Fades From Malus halliana Flowers: Transcriptome Sequencing and DNA Methylation Analysis
Source: Front Plant Sci. 2020 Sep 23;11:576054. doi: 10.3389/fpls.2020.576054 (PMC7539061; doi:10.3389/fpls.2020.576054)
Supplement: Supplementary file 2 [file DataSheet_2.doc]

1 GAAATCGTTA GAAGGTATAA GGTGACATAA ATTCCCCCCT ATTTCTGTTC GAAATCTTCA

61 ATTCTTTAGA TTTAAGATAT TCAATTTTAG GGATATAGGC TTGAAGAATC AATTAGGGAT

121 TTACAAAATG ATTAAAGGGA TTTTGGGTGT TTGCTGTTGC CATTTTTGAA CACAACATCA

181 GTTCCACTAC TCTTTCATTT TCCCTCAATT TCTGAGCAAC CAAAAAAGTA GCATTATTGA

241 CAACATACTG AGCTCCTCGT GTCAACCGTT CTTGAAAGAA TCCTAATAAA GATTTATAGG

301 CAAATTATGC CCTAGAAAAA ATTTAATAAA AAGGACGCTG GACACGTAGG AACCGGCCCG

361 TTTGTAACAG ACTGAGATAG GTCCGGTTCT ATTTCTTAAA AACCCAACAC CCGCTATGTT

421 CCATTTATAA ACGGGTCCGG TCTGGTCCCT TCAACTTTAA GCCCGGCTCG ACTTGTGCCC

481 ACTCCTAAAC TAAACCATAT AAAAACCAAG ATTTCCCTTT CATCTTTCAC ACATATCACG

541 TTACTTTCCA ACGATAATTC AACAATCACA ACAAATAATC AACCATCAAG ATCATATAGT

601 ACTTCACTAA TAAAGACAAC CTTCATATTG TCGTTGTTTT CTACTTGAAA TCCAATTATC

661 TAGCATTGTA ACCCTAAGTT ACAGACACAA ACATAAACTT GAGCAACTTC TATGCATAAG

721 AATCTAGGGT TTTGGACTAA CTCAACAGAA CCTAACAAGA AATAATATTT TGGACCGCTT

781 AACGGAATCC AACGAAGACA AGGTTTCGGA CCACTCAACG GAACAAATAA GGGAAAGGGA

841 TATAAACCAT TCAACGAAAT CCATCTTTAG AATACGCATA GTCCCCCAAT ACGGATTAAC

901 CAAGTGAGAA CATATGCCAT CTGATAGTGT GGTCCCGCGA GACAGATAAC CAAGTAGGAC

961 CACTGATGGT ATAATGTGAC CAAGTAAGCA GTGACCCTAA ATGTAGATTA ACCACGTGGA

1021 GTTAAATTAA CAAGGCTGAA CCACCTATGA AAATAATGTA AGCCTGAAAT CTTAGGAGAG

1081 AATTCTTGCT CTAGGGGACA AATGATTTTC GTACGCCTAA GTGTTTTTTT TAGTGACAGT

1141 AAACTAAGAT TTGAGTACAG AGACATTAAC TGAGATTGAC TCTTGTGAAA GCTTAGTGAG

1201 TTGAAGCACG TAGGCCAATT ATATTGAGCA ATGCGTTAGG TGTAGCGTCT AAACTTCCGT

1261 TGGAGTTTTG TACAGCAATA TAGTGGGGGT GCCGCAAAAT GCAGACAGTA GCAATAAATT

1321 ACGGGCTAGG ATTTTCTCTT CTCTTTTTTT TTCGTTCCAT TCCATCCATT CCTCTCACAT

1381 TCTTTATTTT GTCTTTCTCT TTCTATAAAA AATTAATATA AGATGTTAAT GTAACTTGAC

1441 CGTGACTATT CAAATAGGAG GGGAATGAAG AAGAGGGAAA AAAAGAGAGG AGAGAATCCT

1501 ACTCCGTAAA TTACAAGCAA ACACTTTTTT TTTTTTTTGG ACAAGCAGAA GCAAACAAAC

1561 ACTTGAAAAA GCAGCGAAAG CATGATAAAG GTATCTTATG GTGGTCAAAG ATGTGTGTTG

1621 TAACTAGTTA CACGATTCTG CATTCACATT CATAGAATGT GCTTTTGAAT ATTATATTAC

1681 AGCTAGAGAA TTTTATGCCC TGGGATTGAT TTCCCTTGTC AATGTTGTCG TGCAGAAATG

1741 TTAGACTGGT AGCTATTAAC AAGTTAGACT GGTTAGACTG GTAGCTATTA ACAAGTTAGA

1801 CTGGTAGCTA TTAACAACTG GTAGCTATTA ACAAGTTAGA CTGGTAGCTA TTAACAAGTT

1861 AGACTGTGTG TGTGTGTGTA TTTCACAAGT TAGACTGGTA GCTATTAACA ACTGTTGAAA

1921 TGTATAAACT TGTCAGTGTT TGCTTCTGTG GATATCAGAC ATGCACGTCA CTGGCCTTGT

1981 AAGATTAATT AGGCCGATGG TATCCATAGC GTTAACGTCA TGGCAAACAC ACTCTAATTA

2041 TATATAATGG TAGCTAGGTG TCTTTCTGGA GTCTATGAAG TGGGTAGCAG GCAAAAGATA

2101 AGCTAAGCTT AGCTGCTAGC AGATAAGAG

**Supplementary Figure 2.** The sequence of *MhMYB10* promoter
