# Supplementary figures and images for: How the Color Fades From Malus halliana Flowers: Transcriptome Sequencing and DNA Methylation Analysis
Source: Front Plant Sci. 2020 Sep 23;11:576054. doi: 10.3389/fpls.2020.576054 (PMC7539061; doi:10.3389/fpls.2020.576054)

**
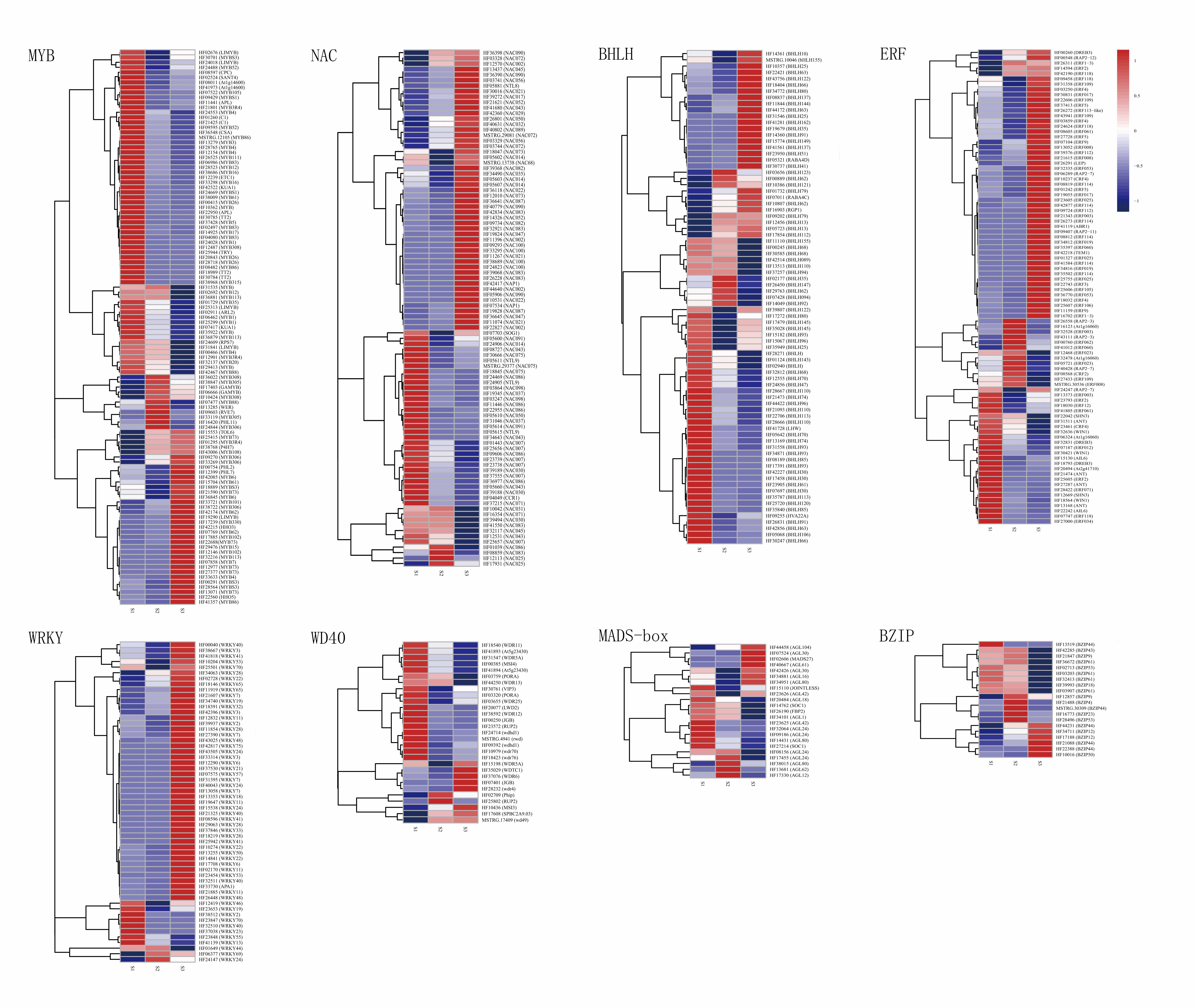
**

**Supplementary Figure S3.** The heatmap of *transcriptional factors in DEGs.*

Supplement: Supplementary file 3 [file DataSheet_3.doc]
