# Supplementary material for: How the Color Fades From Malus halliana Flowers: Transcriptome Sequencing and DNA Methylation Analysis
Source: Front Plant Sci. 2020 Sep 23;11:576054. doi: 10.3389/fpls.2020.576054 (PMC7539061; doi:10.3389/fpls.2020.576054)
Supplement: Supplementary file 4 [file Table_1.docx]

**Table S1. Primers used in this study**

| **Catelogy** | **Primer name** | **Primer sequence (5’-3’)** | **Gene ID/Region** | **Note** |
| --- | --- | --- | --- | --- |
| qRT-PCR analysis | PAL-F | TTTGTGAGGGAGGAGTTGGG | HF01560 | This study |
|  | PAL-R | TTACCCTGGCAGATGGCTTG |  |  |
|  | CHS-F | GGACGAAGTGAGGAGGAAGT | HF00720 |  |
|  | CHS-R | AAGCACAACGGTCTCAACAG |  |  |
|  | CHI-F | GATTCCGTCATCACATTCCAT | HF23861 |  |
|  | CHI-R | TCTTGTCCCACCCAAATACCA |  |  |
|  | F3H-F | TGTAGCGGCTTGTGAGGACTG | HF19324 |  |
|  | F3H-R | TCCGATGGCAAAGCAAAGAAC |  |  |
|  | ANS -F | AGAAGTATGCCAATGACCAGG | HF39612 |  |
|  | ANS -R | AAGTAGTCCTCCCACTCAAGC |  |  |
|  | DFR-F | AGCAGGAACTGTGAATGTGGAG | HF13503 |  |
|  | DFR-R | GAGTTGGGATAATGGTGATGAAAT |  |  |
|  | FLS-F | GACAGACAAGTTACTGGAGGTGC | HF44548 |  |
|  | FLS -R | CGTTCGAGACGAGTAGGGTTAG |  |  |
|  | GSTU17-F | GTTCGGGTGCTTCTTGGG | HF07438 |  |
|  | GSTU17-R | CTCCGGCATGACATCCTT |  |  |
|  | MYB10-F | CCCAGCATTGAGTTAGAGGAAGA | HF36879 |  |
|  | MYB10-R | CCAAAGGTCCGTGCTAAAGG |  |  |
|  | MYB12-F | TACTTATCCAAGCTGGGACTGA | HF28523 |  |
|  | MYB12-R | AAGATAGAAGCCAAGCAACCAC |  |  |
|  | MYB308-F | TTATCCCTACCAAGAAATGACA | HF12487 |  |
|  | MYB308-R | CACAAGCCCACCATTATTACAC |  |  |
|  | MYB114-F | AACCTTCCCATAAAGCATCAG | HF01260 |  |
|  | MYB114-R | GAAACTTGTTCACCCACTCCA |  |  |
|  | MYB111-F | ATTATCTGAGGGCTGACTT | HF26525 |  |
|  | MYB111-R | ATATTCGTTTCCCATCTTG |  |  |
|  | MYB3-F | AAGTGAATGGTGCTGAAAC | HF13279 |  |
|  | MYB3-R | TACGGATAACTGCAAAGCT |  |  |
|  | bHLH3-F | TTCCACTCCTCAAACCTCCTA | HF02940 |  |
|  | bHLH3-R | TTCTTCGTCCTCATCGTCTTC |  |  |
| MYB10 promoter isolation (EU518249) | MYB10P1-F | GAAATCGTTAGAAGGTATAAGG | -2029 to -1229 | Telias et al. 2011 |
|  | MYB10P1-R | TTCGTTGGATTCCGTTAAGC |  |  |
|  | MYB10P2-F | GCTTAACGGAATCCAACGAA | -1248 to -658 |  |
|  | MYB10P2-R | AATGGATGGAATGGAACGAA |  |  |
|  | MYB10P3-F | TTCGTTCCATTCCATCCATT | -677 to 47 | Telias et al. 2011 |
|  | MYB10P3-R | AGTCCAGGCACCTTTTCTCA |  |  |
| Methylation analysis (BSP) | R1 | F: GAAATAGTTTGAAGGTATAAGG  R: ACAACAAACACCCAAAATCC | -2129 to -1973bp | Telias et al. 2011 |
|  | R2 | F: AATGATTAAAGGGATTTTGGGTGTT  R: CGGACCTATCTCAATCTATTACAAA | -2003 to -1745bp | Telias et al. 2011 |
|  | R3 | F: TTTAATAAAAAGGACGCTGGACACG  R: AGTACTATATGATCTTGATGGTTGA | -1808 to -1527bp |  |
|  | R4 | F: TTGTGCCCACTCCTAAACTAAACCA  R:AGTTAGTCCAAAACCCTAGATTCTT | -1657 to -1388bp |  |
|  | R5 | TATGYATAAGAATYTAGGGTTTTGGA  CTTACTTRRTCACATTATACCATCA | -1419 to -1142bp |  |
|  | R6 | F: TTTAGAATACGCATAGTCCC  R:TTTCATAGGTGGTTCAGC | -1264 to -1078bp |  |
|  | R7 | F: GGYTGAAYYAYYTATGAAAATAATG  R: ARACRCTACACCTAACACATTRCT | -1096 to -879bp |  |
|  | R8 | F: TTTTTGTGAAAGCTTAGTGAGTTGAAG  R: CTTCTTCATTCCCCTCCTATTTAA | -950 to -656bp | Telias et al. 2011 |
|  | R9 | F: TTAAATAGGAGGGGAATAAAGAAG  R: TCATACTTTCACTACTTTTTCAAAT | -679 to -536bp |  |
|  | R10 | F: GAAAAAGTAGTGAAAGTATGA  R: AAAAATCAATCCCAAAACATA | -564 to -419bp | Telias et al. 2011 |
|  | R11 | F: TTTCCCTTGTCAATGTTGTCGT  R: GTGACGTGCATGTCTGATATCC | -419 to -158bp |  |
|  | R12 | F: GTTTTAAATTTGTTAGTGTTTGT  R: TCCACTTTCCCTCTCCATAA | -208 to 106bp |  |
