# Supplementary material for: How the Color Fades From Malus halliana Flowers: Transcriptome Sequencing and DNA Methylation Analysis
Source: Front Plant Sci. 2020 Sep 23;11:576054. doi: 10.3389/fpls.2020.576054 (PMC7539061; doi:10.3389/fpls.2020.576054)
Supplement: Supplementary file 7 [file Table_4.docx]

**Table S4. Candidate genes associated with anthocyanin metablism in *Malus halliana***

| **Name** | **Target description** | **NO. all^a^** | **SD vs IF** | | | **SD vs LF** | | **IF vs LF** | |
| --- | --- | --- | --- | --- | --- | --- | --- | --- | --- |
|  |  |  | **NO. up^b^** | **NO. down^c^** | **NO. up^d^** | | **NO. down^e^** | **NO. up^d^** | **NO. down^e^** |
| *PAL* | phenylalanine ammonia lyase | 5 | 0 | 4 | 0 | | 5 | 0 | 5 |
| *4CL* | 4-coumarateCoA ligase | 6 | 2 | 2 | 1 | | 4 | 0 | 3 |
| *C4H* | cinnamate 4-hydroxylase | 4 | 0 | 1 | 0 | | 4 | 0 | 1 |
| *CHS* | chalcone synthase | 5 | 1 | 2 | 0 | | 5 | 0 | 3 |
| *CHI* | chalcone isomerase | 6 | 1 | 3 | 0 | | 5 | 0 | 5 |
| *F3H* | flavanone 3-hydroxylase | 2 | 0 | 2 | 0 | | 2 | 0 | 1 |
| *DFR* | dihydroflavonol 4-reductase | 1 | 0 | 1 | 0 | | 1 | 0 | 1 |
| *ANS* | anthocyanidin synthase | 3 | 0 | 1 | 0 | | 3 | 0 | 3 |
| *AGT* | anthocyanidin 3-O-glucosyltransferase | 3 | 0 | 1 | 1 | | 2 | 1 | 2 |
| *FLS* | flavonol synthase | 6 | 1 | 1 | 2 | | 3 | 2 | 3 |
| *LAR* | leucoanthocyanidin reductase | 3 | 1 | 2 | 1 | | 1 | 1 | 1 |
| *ANR* | anthocyanidin reductase | 3 | 0 | 1 | 0 | | 2 | 0 | 2 |
| **Total** | **Anthocyanin biosynthesis** | **47** | **6** | **21** | **5** | | **37** | **4** | **30** |
| *GST* | glutathione S-transferase | 5 | 0 | 5 | 1 | | 4 | 1 | 3 |
| *ABCC* | ABC transporter C family | 5 | 1 | 4 | 0 | | 4 | 2 | 3 |
| **Total** | **Anthocyanin transport** | **10** | **1** | **9** | **1** | | **8** | **3** | **6** |
| *LAC* | laccase | 11 | 4 | 7 | 4 | | 6 | 3 | 7 |
| *POD* | peroxidase | 1 | 1 | 0 | 0 | | 0 | 0 | 1 |
| *PPO* | polyphenol oxidase | 6 | 5 | 1 | 3 | | 3 | 4 | 2 |
| **Total** | **Anthocyanin degradation** | **18** | **10** | **8** | **7** | | **9** | **7** | **10** |
| **Total** | **-------** | **75** | **17** | **38** | **13** | | **54** | **14** | **46** |
